# Challenges in the postsurgical recovery of cushing syndrome: glucocorticoid withdrawal syndrome

**DOI:** 10.3389/fendo.2024.1353543

**Published:** 2024-04-12

**Authors:** Catherine D. Zhang, Adriana G. Ioachimescu

**Affiliations:** ^1^ Division of Endocrinology and Molecular Medicine, Medical College of Wisconsin, Milwaukee, WI, United States; ^2^ Department of Neurosurgery, Medical College of Wisconsin, Milwaukee, WI, United States

**Keywords:** glucocorticoid withdrawal syndrome, cushing syndrome, cushing disease, adrenal insufficiency, mild autonomous cortisol secretion (MACS)

## Abstract

Glucocorticoid withdrawal syndrome is a challenging clinical phenomenon that can complicate the postsurgical recovery of Cushing syndrome. It is characterized by physical tolerance and dependence to supraphysiologic glucocorticoid exposure during active Cushing syndrome followed by the abrupt decline in cortisol levels after surgical treatment. The symptoms of glucocorticoid withdrawal often overlap with those of postoperative adrenal insufficiency and can be difficult for patients to cope with and for clinicians to treat. This mini review will discuss the clinical characteristics, pathophysiology, and management of glucocorticoid withdrawal syndrome while highlighting recent data in the field.

## Introduction

Endogenous neoplastic hypercortisolism or Cushing syndrome (CS) may be due to a corticotropin (ACTH)-secreting pituitary adenoma, an ACTH- (and rarely CRH) producing ectopic tumor, or ACTH-independent adrenal nodular disease. Overt CS has an incidence of 1.8-3.2 cases/million/year with 70% representing pituitary CS, 25% adrenal CS, and 5% ectopic CS (1, 2). While adrenal CS is rare, mild autonomous cortisol secretion (MACS)— characterized by abnormal 1-mg dexamethasone suppression test in the absence of classic physical features of CS— is diagnosed in up to 30-50% of patients with adrenal nodules and is increasingly recognized as a frequent cause of hypercortisolism (3–5). Regardless of the etiology, surgery to remove the disease-causing tumor is considered the first-line treatment for CS and results in biochemical remission in 60-80% of patients with pituitary CS and virtually 100% of patients with unilateral ACTH-independent adrenal nodular disease (6–10).

Even when biochemical remission is achieved, the postsurgical recovery from CS can be prolonged and difficult (6, 11). Clinical sequalae of hypercortisolism may be slow to improve or persist. Patients often require glucocorticoid supplementation for postoperative adrenal insufficiency due to the chronic suppression of the hypothalamic-pituitary-adrenal (HPA) axis during active CS. Some patients, however, continue to struggle with symptoms that resemble those of cortisol deficiency despite physiologic or even supraphysiologic glucocorticoid replacement doses. These symptoms may be attributed instead to glucocorticoid withdrawal syndrome (GWS), which results from the rapid reduction in relative glucocorticoid concentrations following treatment of CS. GWS is a separate entity from postoperative adrenal insufficiency and can be a challenge for patients to cope with and for clinicians to treat. In this mini review, we discuss the clinical presentation, pathogenesis, and management of GWS following surgical treatment of endogenous CS while highlighting recently published data. We will not specifically address GWS due to medical treatment of CS or exogenous glucocorticoid use, although many of the same principles may apply.

## Clinical presentation of glucocorticoid withdrawal syndrome

Glucocorticoid withdrawal symptoms may start within days after surgery and persist for weeks to months afterwards (12–14). Distinguishing GWS from adrenal insufficiency can be challenging, as the clinical presentation can be similar and both conditions typically coexist in the postoperative period, **Table 1**. Adrenal insufficiency is diagnosed postoperatively by either low basal serum cortisol and/or abnormal cosyntropin stimulation test, and treatment focuses on providing physiologic doses of glucocorticoid replacement (6). GWS, on the other hand, is diagnosed based on characteristic symptoms (i.e., fatigue, malaise, muscle weakness, diffuse myalgias and arthralgias) in the appropriate clinical context (e.g., after surgery for endogenous neoplastic hypercortisolism) and can develop or persist despite physiologic or even supraphysiologic glucocorticoid doses. Other etiologies which may have similar presentations should also be considered, including intercurrent illness requiring an increase in physiologic glucocorticoid replacement and the unmasking of autoimmune disease after resolution of hypercortisolism (15).

**Table 1:** Glucocorticoid withdrawal syndrome versus adrenal insufficiency after surgical treatment of Cushing syndrome.

	Glucocorticoid Withdrawal Syndrome	Postoperative Adrenal Insufficiency
**Diagnosis**	Characteristic symptoms in the appropriate clinical context (e.g., after surgical correction of hypercortisolism).	Biochemical evidence of low basal serum cortisol and/or suboptimal response to cosyntropin stimulation.
**Pathogenesis**	Physical tolerance and dependence to excessive GC levels during active CS followed by abrupt decline in GC exposure after surgery.	Suppressed CRH and HPA axis during active CS followed by postoperative decrease to lower-than-normal GC levels.
**Underlying mechanisms**	Incompletely understood: downregulation of HPA axis and upregulation of inflammatory cytokines and prostaglandins.	Downregulation of HPA axis and sub-physiologic GC replacement.
**Clinical Characteristics**	Similar symptoms as adrenal insufficiency, particularly fatigue, diffuse myalgias, arthralgias, and weakness. * * *Symptoms may occur despite physiologic or supraphysiologic GC replacement and/or normal HPA axis function.*	Flu-like symptoms including fatigue, malaise, anorexia, nausea, vomiting, diffuse myalgias and arthralgias. Possible adrenal crisis with hypotension and altered mental status. *Symptoms occur when physiologic GC requirements are not met.*
**Management**	Patient counseling and reassurance regarding the postoperative recovery process. Slower GC taper and/or temporary increase to the lowest GC dose that controlled symptoms.	Careful evaluation of physiologic GC dose and administration. Increase GC dose for intercurrent illness and use injectable GC when vomiting and unable to take oral medications by mouth.
**Trajectory and Duration**	Worse between 5-12 weeks postoperatively and/or when GC dose tapers below 30 mg hydrocortisone/day. Improvement with HPA axis recovery.	Weeks to years depending on patient and disease-specific factors. Duration shortest for MACS and ectopic CS and longest for overt adrenal CS.

CRH, corticotropin releasing hormone; CS, Cushing syndrome; GC, glucocorticoid; HPA, hypothalamic-pituitary-adrenal; MACS, mild autonomous cortisol secretion.

In a recent longitudinal study of 129 patients with endogenous neoplastic hypercortisolism (59 with MACS, 12 with adrenal CS, 51 with pituitary CS, and 7 with ectopic CS), glucocorticoid withdrawal symptoms were assessed weekly for the first 12 weeks after surgical remission of hypercortisolism and after initiation of glucocorticoid taper for postoperative adrenal insufficiency. The most commonly reported symptoms were myalgias and arthralgias in 50%, fatigue in 45%, weakness in 34%, sleep disturbance in 29%, mood changes in 19%, and headaches in 18% of patients (16). A few symptoms including sleep disturbance and headaches improved, while most symptoms persisted throughout the 12-week study period. Myalgias, arthralgias, and weakness, on the other hand, worsened after postoperative week 5 and were dominant symptoms during postoperative weeks 5-12. These symptoms may contribute to the decline in hand grip strength and patient-reported quality of life that has been observed in the postoperative period (16, 17). However, it is unclear if the trajectory of symptoms observed in the specific study reflect the time course of GWS evolution itself or the glucocorticoid taper utilized in the study. The worsening of myalgias, arthralgias, and weakness symptoms occurred when the glucocorticoid dose tapered below a hydrocortisone equivalent of 30 mg/day, which could be a clinically significant threshold (16). These findings, however, do lend support to the observation that higher glucocorticoid doses help to mitigate glucocorticoid withdrawal symptoms.

Presurgical parameters can be helpful in predicting which patients are more likely to struggle with GWS after surgery. Data suggest that the severity of CS during the active disease phase correlate directly with GWS symptom burden. In a retrospective study of 81 patients undergoing unilateral adrenalectomy for ACTH-independent hypercortisolism, Hurtado et al. found that glucocorticoid withdrawal events were more common and more severe in patients with adrenal CS compared to those with MACS (12). A subsequent prospective study by Zhang et al. identified CS clinical disease severity (defined based on the presence and number of metabolic abnormalities and physical exam findings) as an independent predictor of GWS after surgery for CS in a mixed group of patients (59 with MACS, 12 with adrenal CS, 51 with pituitary CS, and 7 with ectopic CS) (16). Female sex, younger age at the time of CS diagnosis, and decreased proximal muscle strength (as measured by the sit-to-stand test) during active CS were associated with GWS severity on univariable but not multivariable analysis (16).

The degree of HPA axis suppression after surgery also impacts GWS and likely reflects longer standing and/or more severe hypercortisolism during active CS. Hurtado et al. found that GWS events were more frequent and more severe when the 8 am serum cortisol measured 24 hours after the last dose of glucocorticoids was less than 5.0 mcg/dL (< 138 nmol/L). Conversely, patients with 8 am serum cortisol between 5.0 mcg/dL and 10.0 mcg/dL (138 - 275 nmol/L) had fewer GWS symptoms (12). No GWS events were observed when the 8 am serum cortisol measured 24 hours after the last dose of glucocorticoids was greater than or equal to 10.0 mcg/dL (≥ 276 nmol/L), suggesting GWS events lessen as the HPA axis recovers (12). However, GWS has been described in patients with normal HPA axis after surgery as well (18), possibly due to the normalization of the cortisol circadian rhythm after correction of neoplastic hypercortisolism.

## Pathogenesis and underlying mechanisms of glucocorticoid withdrawal syndrome

GWS is a withdrawal reaction characterized by physical tolerance and dependence to excessive glucocorticoid exposure (endogenous or exogenous) and ensues when there is a sudden decline in glucocorticoid concentrations, such as after surgical treatment of CS (19). The mechanisms mediating GWS are complex and incompletely understood. Contributing factors likely include the downregulation of corticotropin-releasing hormone (CRH) and proopiomelanocortin (POMC) —the prohormone to ACTH— and corticotroph atrophy due to chronic suppression of the HPA axis and the upregulation of cytokines and prostaglandins following treatment of hypercortisolism. CRH is also required for the normal functioning of the mesolimbic dopaminergic system, and decreased stimulation of the dopaminergic neurons due to CRH hyposecretion may contribute to GWS symptomatology as well (19).

Although glucocorticoids have potent anti-inflammatory and immunosuppressive effects, patients with both pituitary CS and adrenal-dependent hypercortisolism have been noted to have increased systemic and tissue inflammation markers during active disease compared to healthy controls (20–23). In a prospective observational study of 73 patients with hypercortisolism (63 with MACS, 2 with adrenal CS, and 8 with pituitary CS) and 120 healthy controls, patients with hypercortisolism were found to have alterations in 49/92 inflammatory markers measured including those that are expressed by adipose tissue (20). Following surgical treatment of CS, inflammatory profiles do not normalize (20, 21, 23). Instead, a rise in proinflammatory cytokines, particularly IL-6, has been observed in the early biochemical remission phase and correlates with the development of glucocorticoid withdrawal symptoms (24). In a small study of 17 patients with pituitary CS and 2 patients with adrenal CS, treatment with dexamethasone 0.5 mg/day improved but did not normalize IL-6 levels after surgery, supporting the observation that GWS can persist despite glucocorticoid replacement (24).

In a recent study from the German CS Registry, Vogel et al. studied 80 patients who underwent successful surgical treatment for CS (55 for pituitary CS, 21 for adrenal CS, and 4 for ectopic CS). They found that inflammatory markers remain elevated at 1 year after surgery (compared to the preoperative phase and matched controls without hypercortisolism) and that the proinflammatory state observed after surgery correlated with reduced muscle strength— a key determinant in patent-reported quality of life (25). However, glucocorticoid withdrawal symptoms were not specifically assessed in relation to inflammatory maker levels, and further details about the pathophysiology of GWS needs to be elucidated.

## Management of glucocorticoid withdrawal syndrome

Patient education about what to expect in the postoperative period is crucial in the management of GWS. In a cross-sectional survey study assessing patient (n=341) and clinician (n=54) perspectives following surgical treatment of CS (234 for pituitary CS, 91 for adrenal CS, and 13 for unspecified/unknown etiology), many patients reported feeling unprepared for what happens after surgery and described symptoms consistent with GWS in the open-ended portion of the survey (11). Counseling about GWS should occur before surgery and reviewed frequently at hospital discharge and postoperative follow-up visits, particularly in patients who are at higher risk of GWS symptom burden. Involving family members and friends in the discussion should be encouraged and may increase both patient recall of the information provided and social support during the recovery process (11, 26). In particular, support from family and friends is commonly reported by patients as an useful coping mechanism during CS recovery (11).

After biochemical remission of CS, a glucocorticoid taper is typically utilized to prevent and/or minimize glucocorticoid withdrawal symptoms. However, no standardized guidelines exist to guide glucocorticoid dosing and tapering for GWS, in part due to limited evidence-based data comparing different management strategies. Instead, the approach to glucocorticoid replacement after surgery for CS is derived primarily from clinical experience and expert opinion and should be tailored for the individual patient (14).

While GWS has been described in patients who do not have significant HPA axis suppression, symptoms are likely milder than those with lower cortisol levels after surgery (12, 18). Thus, glucocorticoid replacement is typically reserved for patients with documented biochemical evidence of adrenal insufficiency after surgery. The initial starting dose is often 3- to 4-fold higher than physiologic requirements to try to prevent GWS symptomatology but should be individualized depending on the clinical scenario and risk for GWS. Factors to consider when selecting the starting dose should include clinical and biochemical disease severity during active hypercortisolism, age and sex of the patient, duration of cortisol excess, and comorbidities (16). Patients with MACS typically have milder degrees of cortisol excess compared to overt CS (although the clinical presentation can be heterogenous) and may not require starting doses 3- to 4- times physiologic requirements. In a retrospective study of 108 patients who underwent unilateral adrenalectomy for adrenal-dependent hypercortisolism, no patients developed significant adrenal insufficiency type symptoms when starting doses of hydrocortisone 30 mg per day were used (27).

The type of glucocorticoid used for replacement postoperatively can vary. Hydrocortisone is the most frequently used glucocorticoid (11), and its shorter half-life allows for better replication of the normal cortisol circadian rhythm which may reduce the time to HPA axis recovery. In contrast, longer-acting glucocorticoids, such as prednisone or dexamethasone, may prolong HPA axis recovery but could be more effective at addressing GWS by mimicking the abnormal cortisol circadian rhythm in active CS. For these reasons, when prednisone or dexamethasone is used immediately after surgery, consideration should be given to transitioning to hydrocortisone later in the recovery process. Current limited data, however, have not found significant differences in GWS incidence or outcomes depending on the type of glucocorticoid used (prednisone versus hydrocortisone) (12, 28). The underlying goal of glucocorticoid tapering is to use sufficient doses to prevent significant GWS while avoiding the extended use of excessive doses that may prolong CS manifestations and HPA axis recovery. In our practice, we typically advise patients to taper the glucocorticoid dose by a hydrocortisone equivalent of 5 mg/day every week (starting with the afternoon/evening doses to achieve circadian schedule if all day dosing is initially utilized) until physiologic doses of hydrocortisone 15-20 mg per day is achieved, typically after 6-8 weeks. For intercurrent illness, patients are advised to double their glucocorticoid dose (whenever they are in the tapering schedule) for two to three days and assess response. All patients on glucocorticoid replacement should receive education about sick day rules and provided with a steroid emergency injection kit.

**Figure 1:**
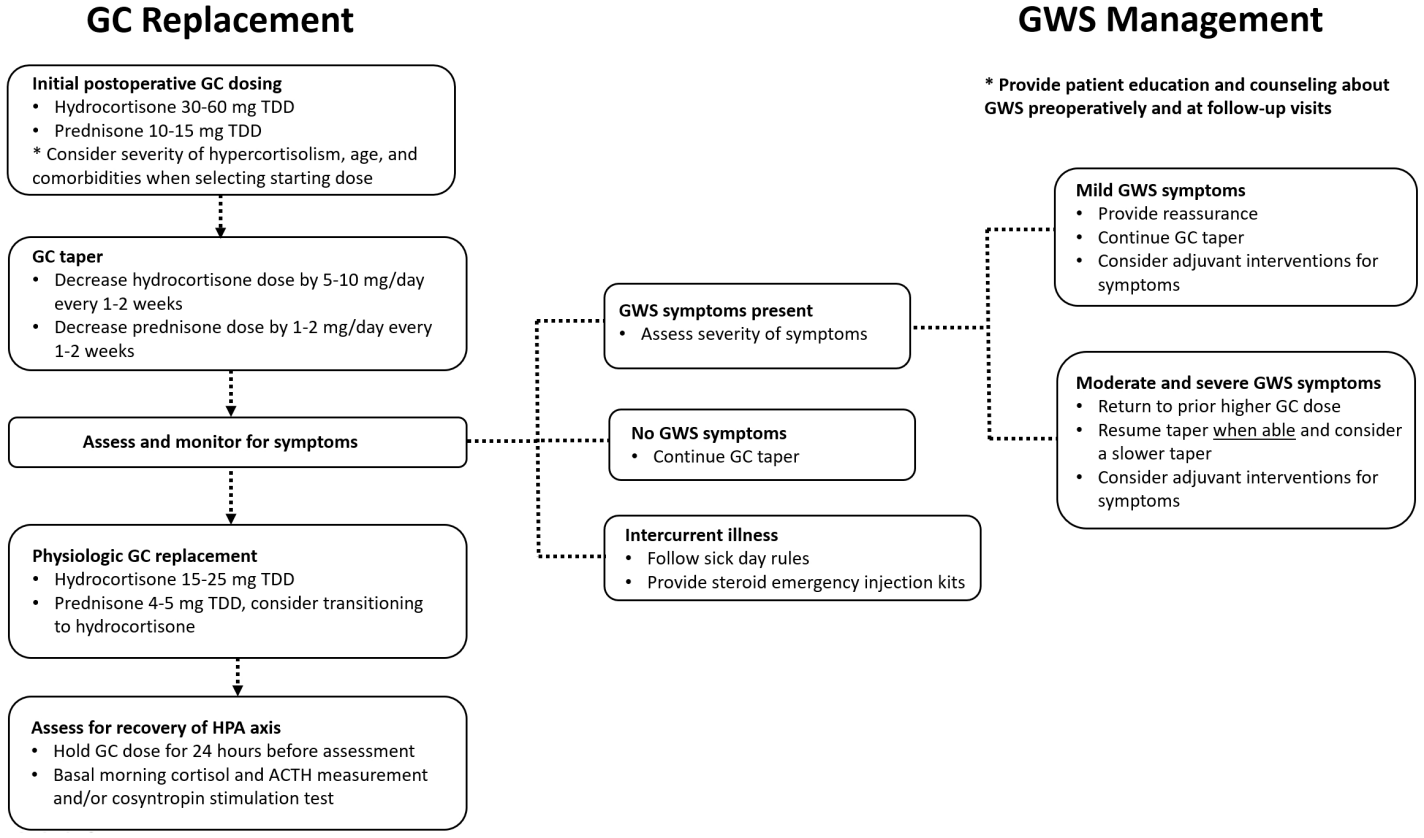
A suggested approach to glucocorticoid dosing and glucocorticoid withdrawal syndrome management after surgical treatment for Cushing syndrome. ACTH, corticotropin; GC, glucocorticoid; GWS, glucocorticoid withdrawal syndrome; HPA, hypothalamic-pituitary-adrenal; TDD, total daily dose.

When a patient develops GWS symptoms during glucocorticoid tapering, management strategies may vary depending on the severity of symptoms, **Figure 1**. If symptoms are mild and tolerable to the patient, reassurance and supportive measures including nonmedical adjuvant treatment can be suggested as applicable. If symptoms are more severe and impair activities of daily living, the glucocorticoid dose could be temporarily increased to the lowest dose that patients felt better on and subsequently tapered more slowly once symptoms improve. It is important to avoid overtreatment with high doses of GC for prolonged periods of time, and the overall rapidity and length of the glucocorticoid taper should be individualized to patient and disease-specific factors and adjusted based on symptoms.

Pharmacotherapy and non-medical ancillary treatments for GWS symptoms can also be utilized as applicable, although clinical data on their efficacy is lacking. This may include warms baths, massage, and short-term use of non-steroid anti-inflammatory medications for myalgias and arthralgias and referral to physical therapy for muscle weakness. For patients with mood symptoms, referral to cognitive behavioral therapy and initiation of antidepressants (in discussion with primary care provider) can be considered. Assessment and replacement of other pituitary hormone deficiencies is warranted in patients following surgery for Cushing disease. Lower postoperative IGF-1 levels have been associated with reduced hand grip strength following surgical remission of CS (29), although the role of GH supplementation in this setting has not been studied.

Finally, full recovery from CS is multifaceted and extends beyond GWS and HPA axis recovery. Acree et al. found that the patient-reported duration of time to complete recovery following surgery for CS was significantly longer than the length of time perceived by clinicians and lasted beyond the duration of glucocorticoid replacement (11). Clinical sequalae of CS may be slow to improve or fail to fully resolve after biochemical remission of CS. In particular, impairments in muscle function and cognitive performance have been reported years after successful treatment of CS and may require targeted interventions to improve long-term outcomes (17, 30, 31).

For patients with persistent and/or recurrent hypercortisolism after surgery, initiation of medical treatment for hypercortisolism can also precipitate GWS, particularly at therapy onset or upon dose increase. In patients experiencing fatigue, nausea, myalgias, and arthralgias on medical therapies for hypercortisolism, the differential diagnosis should include cortisol deficiency from overtreatment, medication-related side effects and/or glucocorticoid withdrawal. Case reports suggest initiation of potent adrenal steroidogenesis inhibitor at low doses with careful up titration to avoid GWS and AI (32), but larger clinical studies are currently lacking.

## Discussion

GWS is a clinical phenomenon that develops after surgical remission of CS due to the rapid decline in cortisol levels. Its symptoms may mimic those of postoperative adrenal insufficiency and can be a difficult to manage. Few evidence-based studies on GWS exist, but emerging longitudinal prospective data suggest that myalgias, arthralgias, and weakness are prominent features of GWS, and that CS severity during active disease is an important predictor of GWS symptom burden (16).

Current challenges in the management of GWS include appropriate recognition of and patient education about glucocorticoid withdrawal symptoms and the lack of robust head-to-head studies comparing different glucocorticoid replacement regimens. Thus, the optimal glucocorticoid starting dose and tapering strategy after surgical remission of hypercortisolism remains unclear, particularly for patients who do not develop postoperative adrenal insufficiency yet may still be at risk of GWS. Future directions for research include mechanistic studies to better understand the pathophysiology of GWS and additional prospective longitudinal studies to evaluate effective management strategies.

## Author contributions

CZ: Writing – original draft, Writing – review & editing. AI: Writing – review & editing.
